# Treatment of Homozygous Type II Antithrombin Heparin-Binding Site Deficiency in Pregnancy

**DOI:** 10.1155/2021/4393821

**Published:** 2021-09-03

**Authors:** Hilde Fiskvik, Anne F. Jacobsen, Nina Iversen, Carola E. Henriksson, Eva-Marie Jacobsen

**Affiliations:** ^1^Department of Hematology, Oslo University Hospital, Oslo, Norway; ^2^Department of Obstetrics and Gynecology, Oslo University Hospital, Oslo, Norway; ^3^Institute of Clinical Medicine, University of Oslo, Norway; ^4^Department of Medical Genetics, Oslo University Hospital, Oslo, Norway; ^5^Department of Medical Biochemistry, Oslo University Hospital, Oslo, Norway

## Abstract

Pregnancy is associated with an increased risk of venous thromboembolism (VTE). Previous VTE and severe thrombophilia are important risk factors. Our case was a 36-year-old woman, gravida 6, para 0, with antithrombin (AT) deficiency caused by a homozygous mutation in the heparin-binding site (HBS). Her history included seven prior VTEs, three early and two late pregnancy losses. She was prophylactically treated with both human plasma-derived AT concentrate (hpATC) and low molecular weight heparin (LMWH), resulting in a successful 6^th^ pregnancy and a healthy live born baby. There is limited evidence and guidance on the management of AT deficiency in pregnancy. Dosing and monitoring of anticoagulants, alone or together with hpATC, must be based on individual risk assessment. The severity of clinical manifestations varies with the type of AT deficiency. Characterization of the AT mutation may aid in the decision-making process and optimize pregnancy outcomes.

## 1. Introduction

AT is a major inhibitor of thrombin (factor (F) IIa), FXa, and several other coagulation factors. Upon binding to heparin, the inactivation rate of AT enhances by 500-1000 [1].

The prevalence of hereditary AT deficiency is 0.02-0.2% in the general population [2] and 1-5% in patients with VTE [3]. AT deficiency can be quantitative (type I), with decreased AT activity and antigen, or qualitative (type II), with decreased AT activity and normal or slightly decreased antigen. Type II AT deficiency is classified into three subtypes based on the site of the causative mutation and the functional consequences, reactive site (RS), HBS, or pleiotropic effects. Heterozygous type II AT RS deficiency is reported to have the greatest potential for thrombosis, and heterozygous type II AT HBS deficiency the least. However, case publications and small studies of patients with homozygous HBS mutations imply that this subgroup is associated with an extreme high risk of thrombosis and pregnancy complications [4–10].

## 2. Case Presentation

A 36-year-old Serbian woman was referred to our hospital with a history of multiple VTEs and pregnancy losses.

At 17 years, she was diagnosed with a right iliac deep vein thrombosis shortly after starting with combined oral contraceptive pills. Investigation of thrombophilia showed reduced AT activity (32%). There was no family history of AT deficiency or VTE. She was treated with warfarin for one year.

At 21 years, she had an extensive left leg thrombosis. Due to substantial symptoms not subsiding on unfractionated heparin (UFH), hpATC was added. She was discharged on warfarin.

At 22 years, she was pregnant for the first time. LMWH was started with fraxiparine 3800 IU daily from gestational week (GW) 5. While on fraxiparine, she experienced an unexplained intrauterine fetal death (IUFD) in GW 23, and postpartum, she developed VTE. At 28 years, she was pregnant for the second time. LMWH with dalteparin 5000 IU × 1 was immediately started, but in GW 9, she suffered a miscarriage. Pregnant for the third time at 29 years, dalteparin 5000 IU × 2 was combined with hpATC 1500 IU twice weekly from GW 8. In GW 22, she experienced IUFD, and eight days postpartum, she was admitted with portal vein and superior mesenteric vein thrombosis. HpATC was administered for five days, warfarin was bridged with dalteparin, and she was discharged with an INR target of 2.5. One month later, she was readmitted with extensive thrombosis in the left arm and was treated with hpATC (2000 IU × 3/weekly). INR was 2.0 on admission, and warfarin was intensified to an INR target of 3.5.

At 30 years, she was pregnant for the 4^th^ time. While on dalteparin 5000 IU × 2 and aspirin, she experienced another early pregnancy loss in GW 11, followed by a thrombosis in left vena saphena magna.

Two years later, she was pregnant for the 5^th^ time and visited our hospital for the first time. Her previous history was not well documented, AT activity was 38 IU/dL (measurement based on FIIa inhibition), and she was using dalteparin 5000 IU × 1 and aspirin. She once again suffered a miscarriage in GW 11. One week later, she was readmitted and diagnosed with portal vein thrombosis.

At 34 years, she was pregnant for the 6^th^ time and was switched from warfarin to dalteparin 100 IU/kg × 2/day (7500 IU × 2) in GW 5. She was diagnosed with a homozygous mutation in exon 2, LEU131Phe, i.e., a type II AT HBS deficiency. HpATC was administered 2000 IU twice weekly. Due to consistently low AT activity around 40 IU/dL, the dose was increased to 2000 IU three times a week from GW 21 and further increased to 2500 IU × 3/weekly from GW 33 (equivalent to 32 IU/kg). LMWH with dalteparin was also increased to 130 IU/kg × 2/day (10 000 IU × 2) in the third trimester.

She was admitted at week 36 + 1 due to contractions. She was not in active labour, and a caesarean section (CS) was scheduled the following morning. The day before the CS, hpATC 3000 IU × 1 was administered and continued for five days. The target AT activity was 80 IU/dL but reached only 64-78 IU/dL in the days around delivery. A healthy baby was delivered, and warfarin was restarted the day after CS with dalteparin bridging. Four days after delivery, the dose of hpATC was reduced from 3000 IU daily to 2000 IU × 3 weekly. Prophylactically, treatment with hpATC continued for 3 weeks postpartum due to her previous history of postpartum VTEs.

## 3. Discussion

Homozygous AT deficiency is not considered compatible with life except for homozygous HBS mutations which are characterized by arterial and venous thrombosis at young age and pregnancy complications [5–8, 10–12]. Our patient had seven VTEs and six pregnancies, before she succeeded, confirming the very high risk.

There are no randomized trials or guidelines on how to manage patients with HBS deficiency in pregnancy, leaving strategies largely based on case reports and expert opinions. Some authors have published guidance based on personal and family history of VTE (15, 21, 23). We argue that the characterization of the subclass of AT deficiency, type, and site of mutation must be considered when deciding prophylaxis and treatment in pregnancy.

The most common indications for the use of AT concentrate in high-risk pregnant AT deficient women are management of VTE that occurs despite treatment with anticoagulants and prophylactic use in the peripartum period.

AT activity of 80-120 IU/dL has been recommended as target during therapeutic substitution with hpATC [13], but for prophylactic use in pregnancy, there are no guidelines on target AT activity.

In most reports on successful pregnancies in homozygous HBS patients [6, 8, 10, 14], patients received both hpATC and anticoagulants.

The obstetric history of our patient made her pregnancy high risk, both for VTE and pregnancy loss, and prompted the use of both hpATC and LMWH. Our patient first received 32 IU/kg twice a week and then three times weekly, and the frequency was based on the biological half-life of hpATC which is about three days. Considering the lack of recommendations for target AT activity for prophylaxis, we chose to substitute with approximately 30 IU/kg. The highest AT activity was achieved in the days around delivery, where AT activity was 64-78 IU/dL.

The anticoagulant effect of LMWH is highly dependent on AT, and low levels of functional AT may lead to heparin resistance and reduced effect of LMWH [3]. It is thought that concomitant administering of hpATC to heparin will overcome the heparin resistance. Routine measurements of anti-FXa activity to guide dosing of LMWH in healthy pregnancy are not recommended [15], and evidence-based guidance to appropriate peak and through levels is limited. However, in high-risk women with AT deficiency, Bramham et al. [16] have suggested monitoring of LMWH with target peak levels of 0,5-1 kIU/L and through levels > 0, 1 kIU/L. In spite of LMWH 260 IU/kg/day and hpATC 32 IU/kg three times weekly, anti-FXa activity in our patient only varied between 0,1 and 0,2 kIU/L (liquid anti-Xa, one-stage chromogenic assay with no exogenous AT). Noteworthy, in a few previous cases of AT deficiency where the aim was to dose-adjust LMWH based on anti-FXa measurements [17, 18], anti-FXa activity was still low despite concurrent administration of hpATC. When VTE occurs after the first trimester despite LMWH 200 IU/day, an alternative to adding hpATC may be to switch to warfarin and return to hpATC and LMWH around delivery. This treatment option has been described successfully in case reports [17, 19, 20]. In the postpartum setting, a rapid transition to a nonheparin anticoagulant is recommended to avoid the problem of heparin resistance. In our patient, with one exception, the VTEs occurred on LMWH.

## 4. Conclusion

AT deficiency is a high-risk thrombophilia, but the risk of VTE in pregnancy varies significantly between the different subclasses. Decisions concerning prophylactic and therapeutic treatment and monitoring of treatment in pregnancy may be facilitated if the type and site of the mutation are known. There is a need for recommendations for both prophylaxis and therapeutic treatment that takes into account the genetic profile, personal and family history of VTE, and obstetric history in each individual patient.
